# Conjugated Linoleic Acid Supplementation Improves Maternal High Fat Diet-Induced Programming of Metabolic Dysfunction in Adult Male Rat Offspring

**DOI:** 10.1038/s41598-017-07108-9

**Published:** 2017-07-27

**Authors:** Stephanie A. Segovia, Mark H. Vickers, Clint Gray, Xiaoyuan D. Zhang, Clare M. Reynolds

**Affiliations:** Liggins Institute, University of Auckland, Auckland, New Zealand

## Abstract

The developmental origins of health and disease hypothesis proposes that an adverse early life environment, including *in utero* exposure to a maternal obesogenic environment, can lead to an increased long-term risk of obesity and related metabolic complications in offspring. We assessed whether maternal supplementation with conjugated linoleic acid (CLA) could prevent some of these adverse effects in offspring exposed to a maternal high fat diet. Sprague-Dawley dams consumed either a: control (CD), control with CLA (CLA), high fat (HF) or high fat with CLA (HFCLA) diet 10 days prior to mating and throughout pregnancy/lactation. Male offspring were weaned onto a standard chow diet. Body composition was quantified by DXA and oral glucose tolerance tests conducted on adult offspring. Gene/protein expression and histological analysis were conducted in adipose tissue. Offspring from HF dams had increased body weight, body fat deposition, impaired insulin sensitivity and adipocyte hypertrophy; all of which were rescued in HFCLA offspring. Molecular and histological analyses of the adipose tissue suggest that disturbances in adipogenesis may mediate the metabolic dysfunction observed in HF offspring. Therefore, CLA supplementation to a maternal obesogenic diet may be a promising strategy to prevent adverse programming outcomes.

## Introduction

Maternal obesity carries an increased risk of adverse metabolic health outcomes for offspring in the long-term^1^. One of the mechanisms proposed to explain how the maternal obesogenic environment programs obesity in offspring is by altering adipogenesis and adipose tissue metabolism in offspring^2^. Increased caloric intake in sheep has been shown to program increased expression of genes related to adipogenesis and lipogenesis in fetal adipose tissue^3, 4^. Human mesenchymal stem cells from infants of obese mothers display enhanced adipogenic potential^5^. These studies suggest that maternal obesity may program an increased propensity for lipid storage in postnatal life.

There is accumulating research aimed at identifying potential early life intervention strategies to prevent adverse developmental trajectories in offspring. Conjugated linoleic acid (CLA) represents the positional and geometric isomers of linoleic acid. It is commonly found in beef and dairy produce of ruminants. CLA is produced as an intermediate in the bacterial biohydrogenation of linoleic acid to stearic acid. Up to 28 different isomers have been identified however the predominant isomer, *c*9, *t*11-CLA is responsible for many of the health effects associated with CLA. It has been reported to influence inflammation, adipocyte differentiation and metabolism in non-pregnant states^6^. The t10, c12-CLA isomer has been associated with the anti-obesity effects of CLA. Administration of a mixture of CLA isomers (predominantly containing equal amounts of the *c*9, *t*11 and *t*10, *c*12 isomers) has been linked to a reduction in fat mass in a number of animal and human studies^7–9^. Studies administering the isomers in isolation suggest that the *t*10,*c*12 isomer is primarily responsible for reducing fat mass^10^, which may be mediated by reducing fat storage in mature adipocytes and therefore reducing adipocyte size^11, 12^. In contrast, the *c*9*,t*11 isomer has been reported to promote adipogenesis through induction of peroxisome proliferator-activated receptor-γ (PPARγ)^13^. This may be considered advantageous as it counteracts adipocyte hypertrophy, which can lead to inflammation and dysregulation of the adipose tissue^14^. However, it must be noted that there is some disagreement in the effectiveness of CLA in human intervention studies. A meta-analysis concluded that although CLA reduced fat mass in overweight/obese individuals, the magnitude was small, and potentially not clinically significant^15^.

We have previously reported that maternal supplementation of CLA to a high fat (HF) diet reversed HF diet mediated metabolic inflammation during pregnancy^16^. CLA supplementation to a HF diet also blunted the adverse early life growth trajectory (including reduced fetal size and accelerated growth in the pre-weaning period) in offspring from HF dams^16^. Female offspring from HF dams had early pubertal onset, and increased fat mass, dyslipidemia and hyperleptinemia in adulthood, all of which were reversed in HFCLA female offspring^17^. However, the long-term effects of maternal CLA supplementation on male offspring health have not been examined. The present study therefore investigated the impact of maternal supplementation with mixed CLA isomers to a control and HF diet on whole-body metabolism, adipocyte morphology and adipose tissue function in adult male offspring.

## Results

### Maternal diet altered postnatal growth in adult male offspring

**Figure 1:**
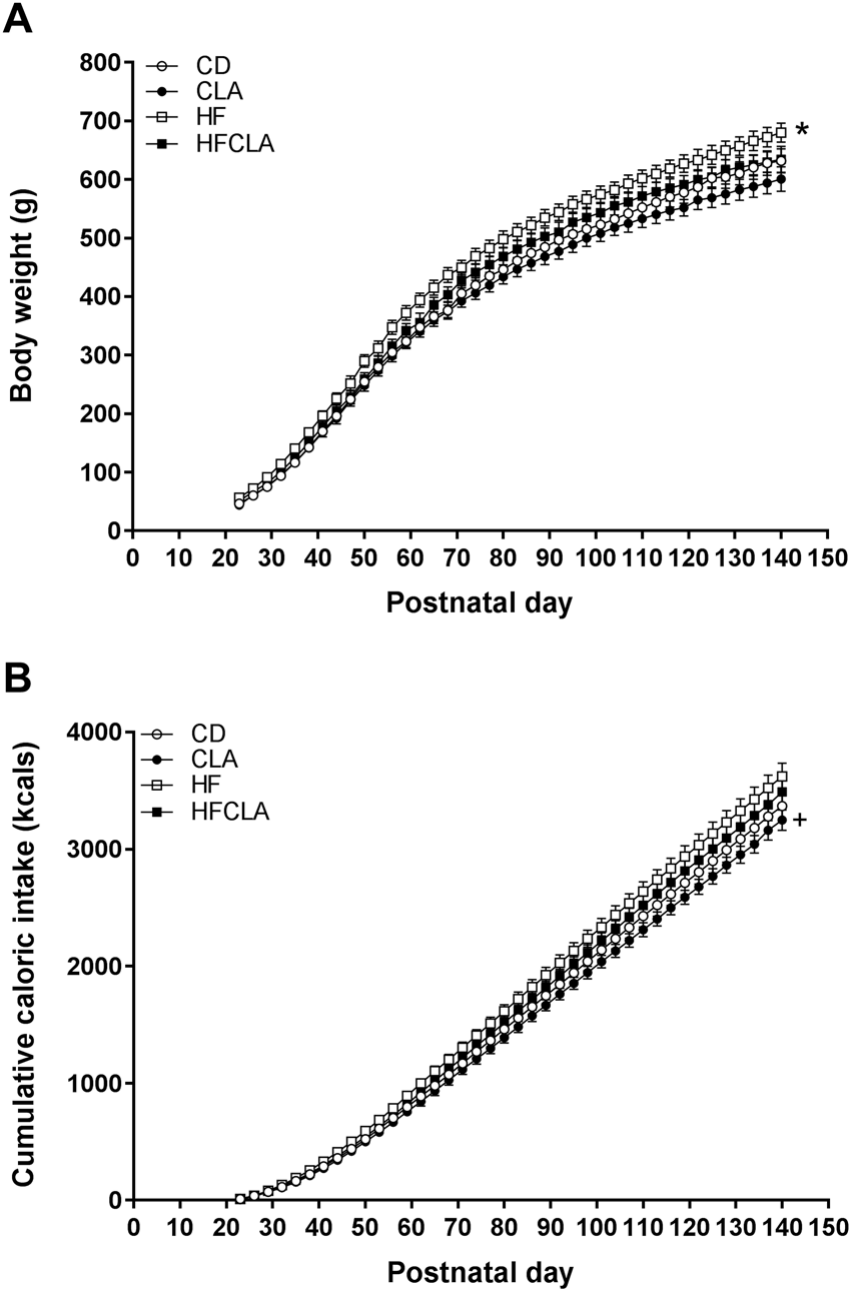
Postnatal growth curve and caloric intake. (**A**) Body weights and (**B)** cumulative caloric intake of male offspring post-weaning. Data analysed by two-way repeated measures ANOVA, with maternal diet and time as factors. *Post-hoc* Holm-Sidak analysis was performed. Data expressed as means ± SEM (*n* = 5–6 litters/group), where *P < 0.05 vs CD and CLA, and ^+^ *P* < 0.05 vs HF.

Offspring from HF mothers were significantly heavier than offspring from CD and CLA mothers, with these differences emerging at P56 (Fig. 1A). While there were no significant differences between weights of offspring from HF and HFCLA mothers from P23-122, significant differences were present from P125-140. When analysed by repeated measures two-way ANOVA, HF offspring had significantly increased cumulative caloric intake compared to CLA offspring throughout the post-weaning period (Fig. 1B). However, total calories consumed throughout the study was not different between groups.

### HF offspring had increased adiposity, which was prevented in HFCLA offspring

**Figure 2:**
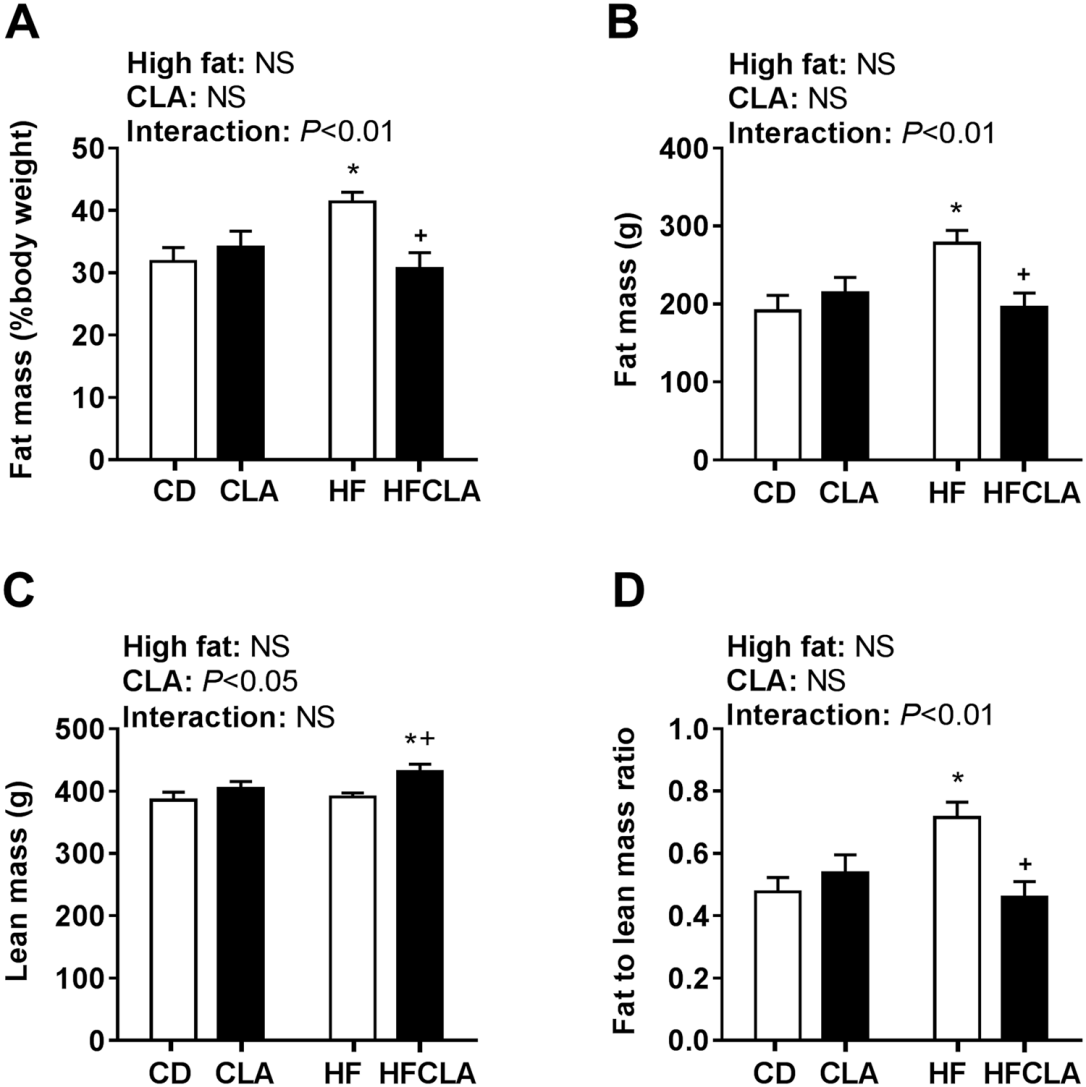
Offspring body composition. Body composition was determined by DXA. **(A)** Fat mass expressed as a percentage of body weight **(B)** fat mass in grams **(C)** lean mass in grams and **(D)** fat mass to lean mass ratio. Data expressed as means ± SEM (*n* = 5–6 litters/group), where *P < 0.05 vs CD and ^+^ *P* < 0.05 vs HF.

DXA scans revealed that HF offspring had significantly greater fat mass when expressed relative to body weight (Fig. 2A) and in absolute terms in grams (Fig. 2B) compared to CD and HFCLA offspring. Further there was a significant effect of maternal CLA supplementation on lean mass in offspring, with *post-hoc* analysis revealing significantly greater lean mass in HFCLA offspring compared to CD and HF offspring (Fig. 2C). There was a significant interaction in the fat to lean mass ratio, with HF offspring having a greater ratio compared to CD and HFCLA offspring (Fig. 2D). At P150, offspring from HF mothers had significantly increased body weight and retroperitoneal adipose tissue percentage compared to all other offspring (Table 1).

**Table 1:** Male offspring body/tissue weights and plasma metabolic profile at P150.

	CD	CLA	HF	HFCLA	Maternal HF	Maternal CLA	Interaction
Body weight (g)	635.0 ± 67.7	600.4 ± 70.1^+^	707.0 ± 51.0*	651.6 ± 63.1^+^	*P* < 0.01	*P* < 0.05	NS
Retroperitoneal adipose tissue (% body weight)	2.7 ± 0.9	2.4 ± 1.1^+^	4.0 ± 0.7*	2.7 ± 1.2^+^	*P* < 0.05	*P* < 0.05	NS
Liver (% body weight)	2.4 ± 0.1	2.5 ± 0.3	2.4 ± 0.2	2.5 ± 0.2	NS	NS	NS
Fasting glucose (mmol/L)	8.55 ± 0.19	8.74 ± 0.19	8.95 ± 0.21	9.03 ± 0.22	NS	NS	NS
Fasting insulin (ng/ml)	1.80 ± 0.29	1.80 ± 0.31	1.27 ± 0.20	2.00 ± 0.31	NS	NS	NS
Leptin (ng/ml)	7.13 ± 1.46	6.93 ± 1.03^+^	13.59 ± 2.06*	8.55 ± 1.19^+^	*P* < 0.01	NS	NS
IL-1β (ρg/ml)	20.37 ± 3.39	30.67 ± 5.65	32.11 ± 8.25	16.14 ± 1.22^+^	NS	NS	*P* < 0.05
IL-10 (ρg/ml)	22.29 ± 2.58	27.99 ± 2.30	34.72 ± 9.12	21.26 ± 1.42	NS	NS	NS
MCP1 (ρg/ml)	727.2 ± 66	788.22 ± 133	778.88 ± 95	775.52 ± 70	NS	NS	NS
Free fatty acids (mmol/L)	0.84 ± 0.14	0.98 ± 0.16	0.81 ± 0.11	0.83 ± 0.15	NS	NS	NS
Triglycerides (mmol/L)	0.80 ± 0.09	0.94 ± 0.09	0.93 ± 0.06	0.88 ± 0.09	NS	NS	NS
ALT (U/L)	52.95 ± 3.83	51.23 ± 2.20	45.38 ± 2.85	48.36 ± 3.33	NS	NS	NS
AST (U/L)	146.87 ± 8.37	134.01 ± 7.45	133.87 ± 11.43	145.27 ± 8.83	NS	NS	NS
Lipase (U/L)	17.19 ± 1.26	17.59 ± 1.22	13.65 ± 0.69*	15.62 ± 1.33	*P* < 0.05	NS	NS
LDL (mmol/L)	0.26 ± 0.02	0.54 ± 0.05*^,+^	0.31 ± 0.03	0.43 ± 0.07	NS	*P* < 0.001	NS
HDL (mmol/L)	1.24 ± 0.08	1.50 ± 0.08*	1.26 ± 0.08	1.42 ± 0.11	NS	*P* < 0.01	NS
Total cholesterol (mmol/L)	1.81 ± 0.09	2.33 ± 0.14*	1.95 ± 0.12	2.14 ± 0.16	NS	*P* < 0.05	NS
LDL/HDL ratio	0.21 ± 0.01	0.36 ± 0.02*^,+,^^	0.23 ± 0.02	0.26 ± 0.03	NS	*P* < 0.01	*P* < 0.05

Data was analysed by two-way ANOVA (*n* = 5–6 litters/group). Data expressed as means ± SEM, where *P < 0.05 vs CD, + *P* < 0.05 vs HF and ^*P* < 0.05 vs HFCLA.

### HFCLA offspring had a normalised insulin response compared to HF offspring when subjected to an oral glucose challenge

**Figure 3:**
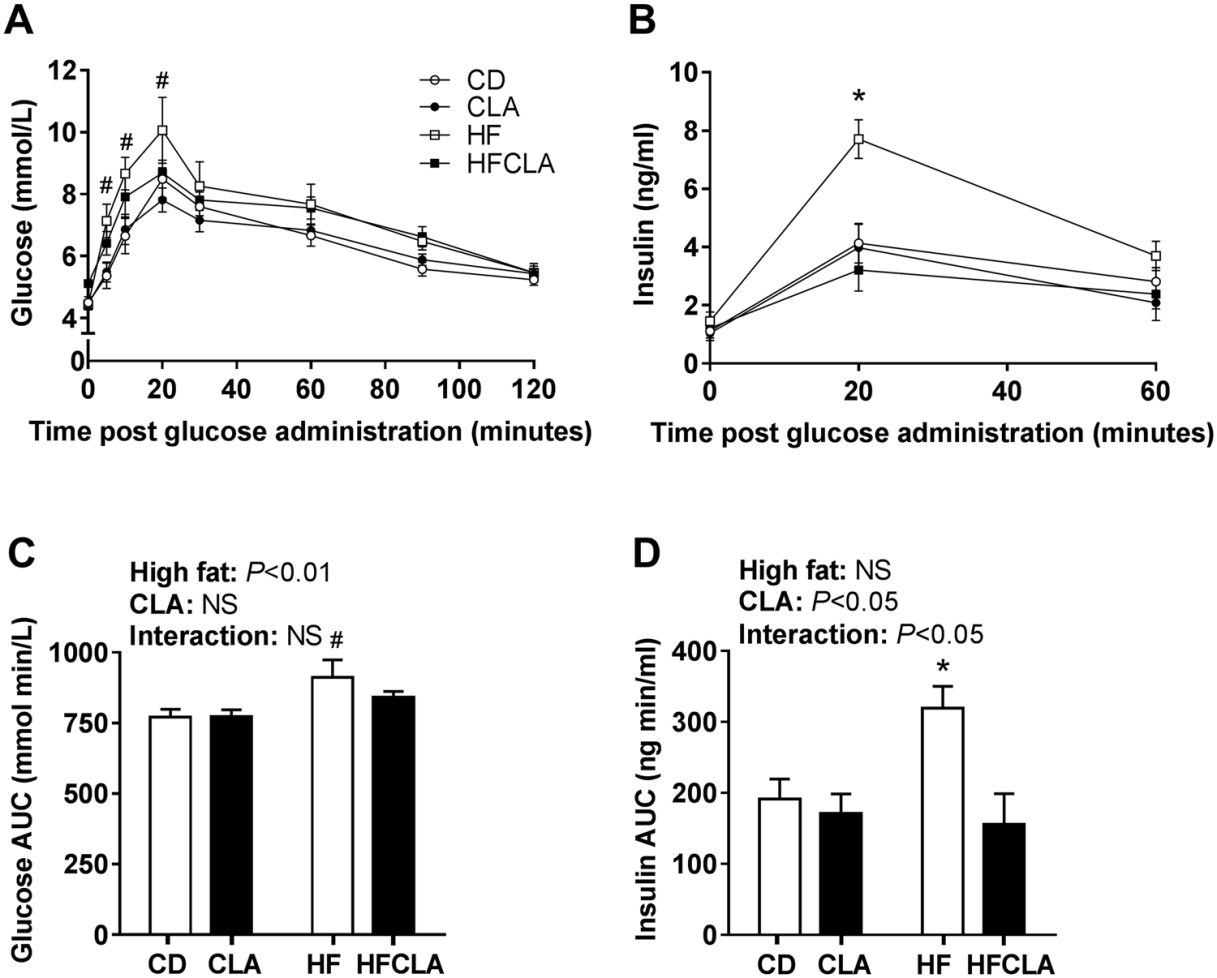
Offspring oral glucose tolerance tests. **(A)** Glucose concentrations and **(B)** plasma insulin concentrations after oral administration of glucose. **(C)** Glucose area under the curve and **(D)** insulin area under the curve. Data expressed as means ± SEM (*n* = 5–6 litters/group), where ^#^ *P* < 0.05 vs CD and CLA and *P < 0.05 vs all other groups.

At 5, 10 and 20 minutes post glucose administration, glucose concentrations were significantly greater in HF offspring compared to CD and CLA offspring (Fig. 3A). This was mirrored by a significant maternal HF effect in the glucose AUC, with *post-hoc* analysis showing a significantly greater AUC in HF offspring compared to CD and CLA offspring (Fig. 3C). At 20 minutes post glucose administration, plasma insulin concentrations were significantly greater in HF offspring compared to all other offspring (Fig. 3B). Similarly, the insulin AUC was significantly increased in HF offspring compared to all other offspring (Fig. 3D).

### Male adult offspring plasma profile suggests benefits of maternal CLA supplementation to a HF diet, but possible impairments when supplemented to a CD diet

The plasma metabolic profile of male offspring at P150 is presented in Table 1. Despite differences in oral glucose tolerance, there were no significant differences in fasting glucose or insulin concentrations. However, fasting leptin concentrations were significantly greater in HF offspring compared to all other groups. There was a significant interaction in the concentration of IL-1β, with a reduction in HFCLA offspring compared to HF offspring. There were no significant differences in IL-10 or MCP1 concentrations. There were no significant differences in free fatty acids, triglycerides, ALT or AST. There was a significant effect of a maternal HF diet on lipase concentrations, with a significant reduction in HF offspring compared to CD. There was a significant effect of maternal CLA supplementation on LDL, HDL and total cholesterol. When expressed as a LDL/HDL ratio, there was a significant interaction, with an increase in CLA offspring compared to all other groups. Hepatic markers relevant to blood lipid concentrations, inflammation and glucose metabolism did not demonstrated differences between groups (Supplemental Fig. 1).

### Divergent effects of maternal HF and HFCLA diets on adipocyte morphology and adipogenic markers in adult male offspring

**Figure 4:**
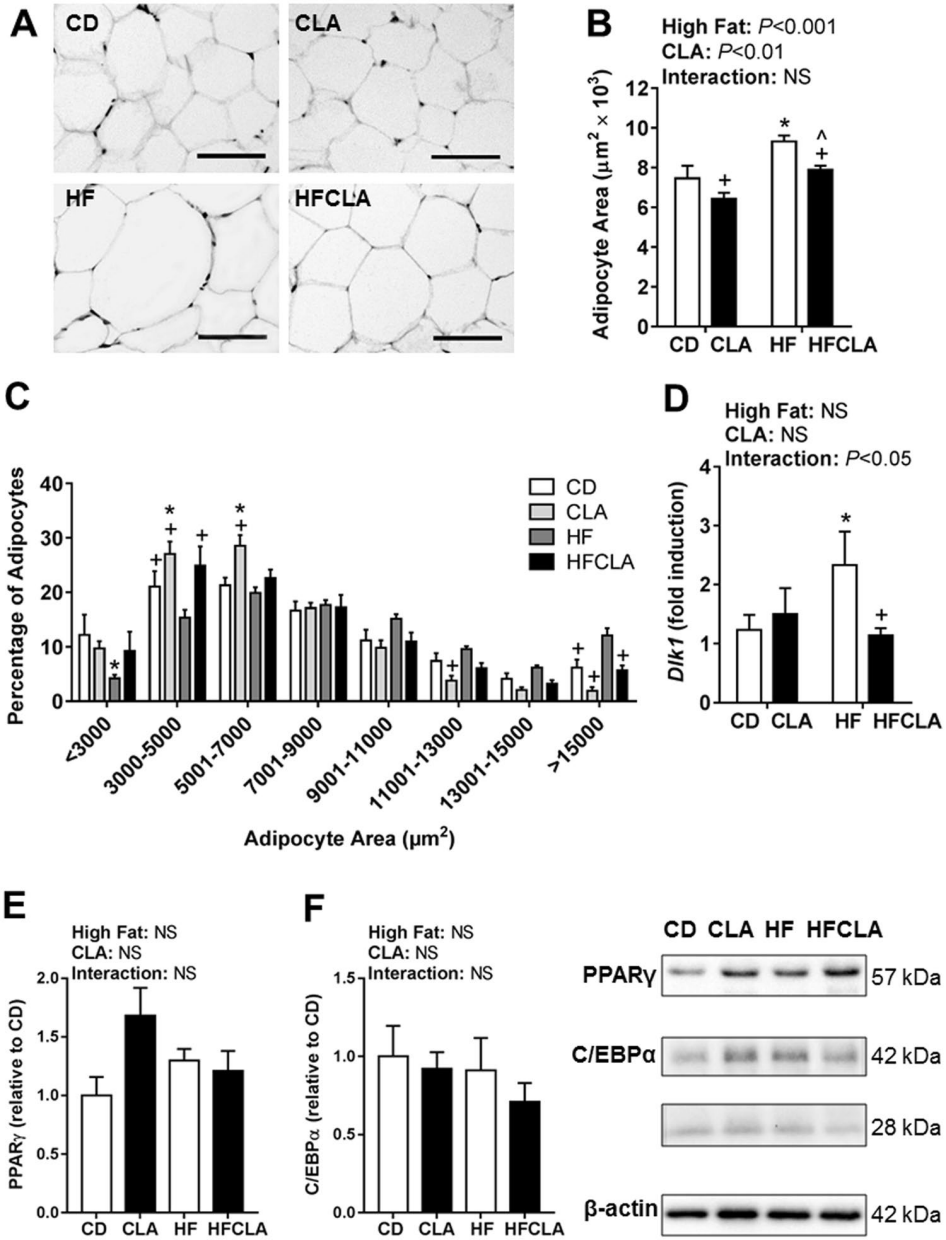
Retroperitoneal adipose tissue histology and expression of adipogenic markers. (**A**) Representative adipocyte histological images of adipose tissue sections (scale bar represents 100 µm). **(B)** Average adipocyte size and **(C)** adipocyte size distribution (*n = *5–6 litters/group). **(D)** *Dlk1* expression determined by qPCR (*n = *5–6 litters/group). Cropped versions of representative western blots of adipogenic regulators **(E)** PPARγ and **(F)** C/EBPα (*n* = 5 litters/group) are presented. Data expressed as means ± SEM, where *P < 0.05 vs CD, ^*P* < 0.05 vs CLA and ^+^ *P* < 0.05 vs HF.

On average, HF offspring had significantly larger adipocytes compared to all other offspring and CLA offspring had a significantly smaller average adipocyte size compared to HFCLA offspring (Fig. 4B). The distribution of adipocyte size differed between groups (Fig. 4C). HF offspring had significantly less small adipocytes between the size of 3000-5000 µm^2^ and more large adipocytes greater than 15000 µm^2^ compared to all other offspring. Gene expression of *Dlk1*, a pre-adipocyte marker was significantly upregulated in HF offspring compared to CD and HFCLA offspring (Fig. 4D). However, PPARγ and C/EBPα protein expression were unchanged between groups (Fig. 4E,F).

### Differential impact of maternal diets on gene expression in the retroperitoneal adipose tissue

**Figure 5:**
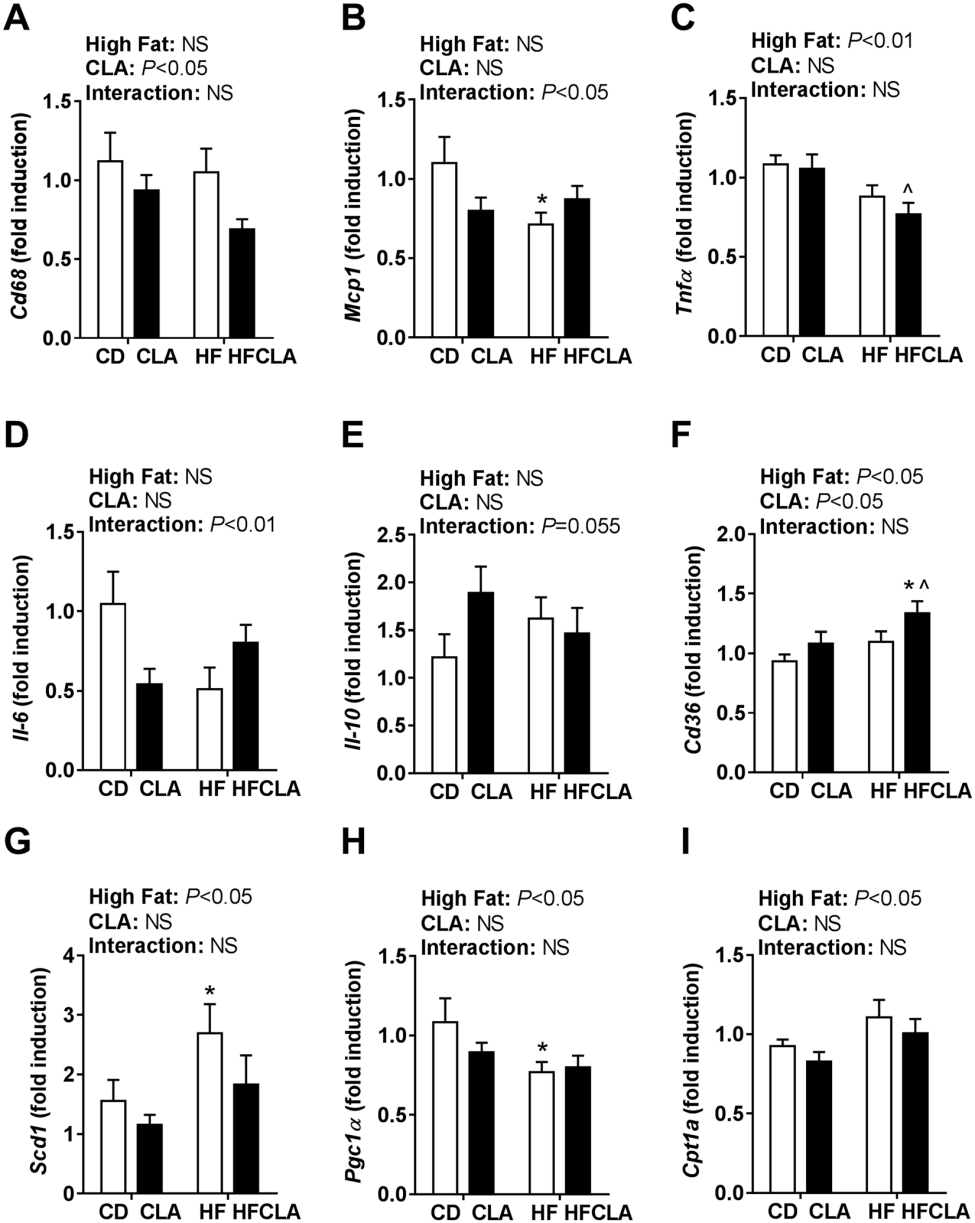
Gene expression in the retroperitoneal adipose tissue of adult male offspring. qPCR was performed to determine gene expression of **(A)** *Cd68*; **(B)** *Mcp1*; **(C)** *Tnfα*; **(D)** *Il-6*; **(E)** *Il-10;* **(F)** *Cd36;* **(G)** *Scd1*; **(H)** *Pgc1α* and **(I)** *Cpt1a*. Data analysed by two-way ANOVA (*n* = 5–6 litters/group). Data expressed as mean ± SEM, where *P < 0.05 vs CD and ^*P* < 0.05 vs CLA.

There was a significant effect of maternal CLA supplementation on reducing the expression of the macrophage marker *Cd68* (Fig. 5A). In contrast, *Mcp1* expression was significantly reduced in HF offspring compared to CD offspring (Fig. 5B) and there was a significant maternal HF effect on reducing *Tnfα* expression (Fig. 5C). There were no significant differences in *Il-1β* expression (data not shown). There was a significant interaction in *Il-6* expression (Fig. 5D) and strongly trending interaction (*P* = 0.055) in *Il-10* expression (Fig. 5E). Expression of the key fatty acid transporter *Cd36* was significantly increased in HFCLA offspring compared to CD and CLA offspring (Fig. 5F). *Scd1*, an enzyme that catalyses the reaction that metabolises saturated fatty acids to monounsaturated fatty acids (MUFAs), was significantly increased in HF offspring compared to CD offspring (Fig. 5G). There was significant down-regulatory effect of a maternal HF diet on expression of *Pgc1α*, a transcriptional co-activator which regulates glucose metabolism (Fig. 5H). There was a significant effect of a maternal HF diet on up-regulation of expression of *Cpt1α*, an enzyme involved in β-oxidation which has beneficial effects on adipose tissue insulin sensitivity (Fig. 5I)^18^.

### Macrophage-related gene expression in the SVF was partially improved in HFCLA offspring compared to HF offspring

**Figure 6:**
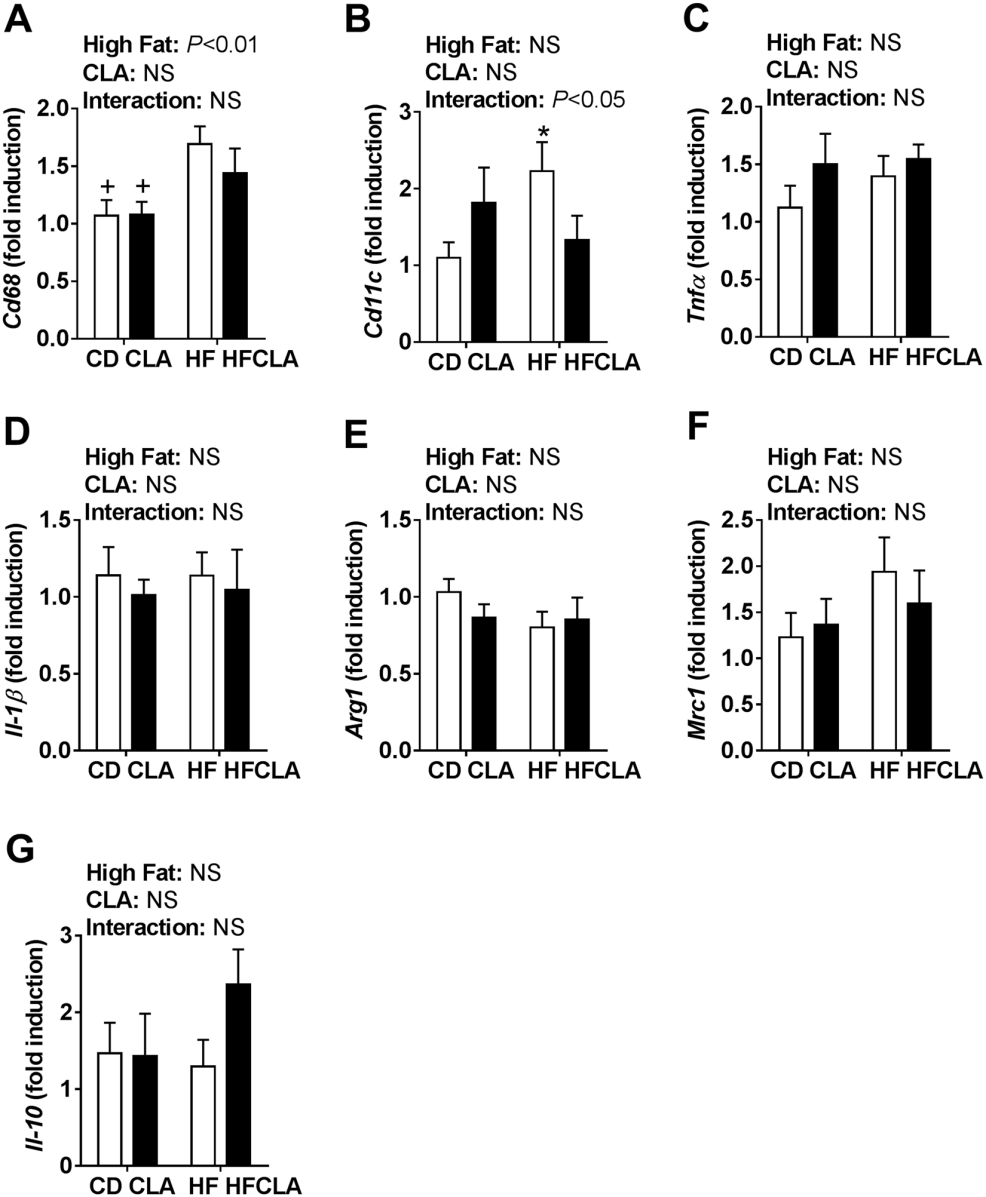
Gene expression in the stromal vascular fraction from gonadal adipose tissue. qPCR was performed to determine gene expression of **(A)** *Cd68*; **(B)** *Cd11c*; **(C)** *Tnfα*; **(D)** *Il-1β*; **(E)** *Arg1*; **(F)** *Mrc1* and **(G)** *Il-10*. Data analysed by two-way ANOVA (*n* = 5–6 litters/group). Data expressed as mean ± SEM, where *P < 0.05 vs CD and ^+^ *P* < 0.05 vs HF.

There was a significant effect of a maternal HF diet on the expression of *Cd68* (Fig. 6A). There was a significant increase in HF offspring compared to CD and CLA offspring, with expression in HFCLA offspring being intermediate. There was a significant interaction in *Cd11c* expression (Fig. 6B), a cell surface marker expressed on M1 macrophages. HF offspring had significantly increased expression compared to CD offspring. However, there were no significant differences in expression of the pro-inflammatory cytokines *Tnfα* and *Il-1β* (Fig. 6C,D). Expression of *Arg1, Mrc1* and *Il-10*, genes induced by M2 macrophages, was not different between groups (Fig. 6E–G).

## Discussion

We have previously reported the impact of maternal CLA supplementation to either control or obesogenic diets on maternal, fetal, neonatal and adult female offspring outcomes^16^. In the present study, we assessed male offspring for long-term growth and metabolic profiles. At P150, HF offspring had significantly increased body weight compared to all other offspring. This increased body weight cannot be fully explained by alterations in food intake, as HF offspring did not consume significantly more cumulative calories than all other offspring at P140. This suggests that HF offspring may have an inherent predisposition to store increased body fat. Indeed when we assessed body composition at P140 by DXA and at P150 using retroperitoneal adipose tissue percentage as a proxy, HF offspring had increased fat percentage compared to all other offspring. In line with this, HF offspring also displayed increased circulating leptin concentrations and an impaired insulin response compared to all other offspring. Thus, HF offspring had programmed obesity and metabolic impairments which were prevented in HFCLA offspring.

In addition to having increased body fat percentage, HF offspring had significantly increased average adipocyte size compared to all other offspring, with a greater proportion of larger adipocytes compared to all other offspring. *Dlk1* is expressed by pre-adipocytes and acts as an inhibitor of adipogenesis^19^. Therefore, it is in agreement with the histological data that adipose tissue expression of *Dlk1* was significantly upregulated in HF offspring only. This may indicate that normal adipogenesis in HF offspring is blunted, and thus the differentiated adipocytes in these offspring have become hypertrophic to cope with energy storage demands^20^. Taken together, our findings suggest that HF offspring have a diminished capacity for healthy adipose tissue expansion, which may contribute to the adipose tissue dysfunction observed. PPARγ and C/EBPα positively regulate each other’s expression to stimulate adipogenesis^21, 22^. Although we did not detect significant differences in protein expression, this may be due to their regulation by phosphorylation^23, 24^.

Following this, we observed a reduction in circulating IL-1β in HFCLA offspring compared to HF offspring. IL-1β has been shown to limit adipose tissue expansion and thus potentially favour liver steatosis and insulin resistance (IR)^25^. We further assessed the adipose tissue inflammatory phenotype by examining gene expression of macrophage markers and cytokines in both the retroperitoneal adipose tissue and SVF. In obesity, there is an infiltration of pro-inflammatory M1 activated macrophages, which are positive for CD11c, into the adipose tissue, mainly arising from circulating monocytes originating from the bone marrow^26–28^. Secretion of MCP1 is considered to be a major driver of macrophage infiltration^29^. In contrast, in lean adipose tissue, macrophages are mainly M2 activated (indicated by increased expression of *Il-10, Mrc1* and *Arg1*), and are confined to the interstitial space^30^. In the obese state, these M2 activated macrophages remain in the interstitial space but become overrun by infiltrating M1 activated macrophages^28^. In obesity, macrophage infiltration precedes an increase in circulating insulin concentrations^31^, implicating adipose tissue macrophages and the induction of inflammation as potential causative factors of IR. In the present study, there was a reduction of the general macrophage marker *Cd68* in the adipose tissue of offspring from CLA supplemented mothers. In the SVF, there was increased expression of *Cd68* and *Cd11c* in HF offspring, with a partial reduction in HFCLA offspring. In the absence of impaired insulin sensitivity in these animals, the alterations in macrophage and polarisation markers may reflect early evidence of an unfavourable adipose tissue environment, not necessarily accompanied by increased inflammation at this point. Indeed, Pang *et al*. has demonstrated that infiltrating macrophages in obesity may contribute to adipose tissue remodelling by promoting angiogenesis^32^, and thus may be a compensatory defence against adipose tissue hypoxia. We did not observe differences in the M2 markers *Arg1* and *Mrc1*, which is in agreement with Lumeng *et al*.^28^.

CD36 is involved in uptake of long-chain fatty acids, and increased adipose tissue expression of CD36 is associated with obesity in humans^33^. However, in more recent years, a more complex role of CD36 has emerged. CD36 may play an important role for adipogenesis, as it has been shown to be increased during *in vitro* differentiation of pre-adipocytes and to be correlated to PPARγ expression^34^. Furthermore, CD36 knockout mice are resistant to HF diet induced increases in subcutaneous and gonadal adipose tissue mass due to reduced fatty acid uptake^34^. However, CD36 knockout mice also have reduced adipocyte differentiation due to smaller adipocytes, ectopic hepatic triglyceride deposition and increased IR^35^. The increased expression of *Cd36* observed in HFCLA offspring may indicate an increased shuttle of lipids into adipocytes, which would be more favourable than ectopic storage.

SCD1 is the enzyme responsible for metabolising saturated fatty acids into MUFAs^36^. SCD1 is significantly increased in morbidly obese individuals with IR^37^. Findings by Carobbio *et al*. suggest that the increased expression of *Scd1* in the adipose tissue is an adaptive response to maintain lipid desaturation under the demands of obesity^38^. Therefore the increased *Scd1* expression we observed in HF offspring is likely a compensatory response reflective of a state of metabolic stress in the adipose tissue. A number of other programming studies have demonstrated increased expression and/or activity of SCD1 in offspring exposed to adverse maternal environments, including a HF and sucrose diet^39^, HF diet^40^ and maternal IR^41^. However, these studies only investigated SCD1 in the liver. A study by Holness *et al*. suggests that increased *Scd1* expression in adipocytes from offspring exposed to a maternal low protein diet may partially contribute to the development of obesity in this paradigm^42^.

PGC-1α is a key transcriptional regulator and promoter of mitochondrial biogenesis^43^. Insulin resistant individuals have reduced mRNA and protein expression of PGC1 in their adipose tissue^44^. Moreover, adipose tissue-specific PGC-1α knockout mice have reduced adipose tissue expression of mitochondrial and thermogenesis markers at an ambient temperature and impaired hepatic insulin sensitivity when challenged with a HF diet^45^. These findings implicate PGC-1α as a key regulator of glucose homeostasis and metabolism. Furthermore, during adipocyte differentiation, there is a robust increase in mitochondrial genes^46^ and inducing mitochondrial dysfunction *in vitro* suppresses adipocyte differentiation in pre-adipocytes^47^. In the present study, HF offspring had reduced adipose tissue expression of *Pgc1α* compared to CD offspring, which may explain the impaired insulin sensitivity concomitant with impaired adipogenesis in these animals. CPT1 is a mitochondrial enzyme, responsible for catalysing the reaction that permits the shuttle of fatty acids into the mitochondria for β-oxidation^48^. The three isoforms of CPT1 are differentially expressed depending on the tissue; their roles are more well defined in the liver and muscle compared to the adipose tissue^48^. We observed a significant main effect of a maternal HF diet on increasing offspring adipose tissue expression of *Cpt1a*, which may represent a compensatory mechanism, as CPT1A overexpression in adipocytes promotes fatty acid oxidation and thus improves insulin sensitivity^18^.

There are some interesting alterations to note in CLA offspring, which highlight the need for caution in supplementation during pregnancy. Interestingly, CLA offspring had the lowest body weight and cumulative caloric intake, although it was not significantly lower than CD offspring. Although there was a significant effect of maternal CLA supplementation on increasing circulating cholesterol concentrations, there were no changes in expression of genes involved in cholesterol metabolism (*Ldlr, Abca1, Abgc8*) in the liver. This may be reflective of a reduced triglyceride storage capacity in CLA offspring, who had the smallest average adipocyte size despite similar fat percentage to other offspring.

## Conclusion

The present study demonstrated a beneficial effect of maternal CLA supplementation to a HF diet on physiological, metabolic and adipogenic markers in adult male offspring. Maternal CLA supplementation to a HF diet improved maternal inflammatory status, independent of changes in body weight and glucose/insulin metabolism. Therefore, we expected altered inflammatory regulation in HF offspring. However, cytokine expression was largely unaltered in offspring, although increased expression of macrophage markers may indicate an increased inflammatory potential if faced with an additional postnatal challenge. The most pronounced alterations were in adipocyte morphology and markers related to adipogenesis. Our findings therefore suggest that impaired adipogenesis is a key mechanism mediating the programming of obesity and metabolic dysfunction.

## Research Design and Methods

### Animal model

All animal experiments were approved by the Animal Ethics Committee at the University of Auckland and were performed in accordance with relevant institutional guidelines and procedures. Animal procedures were carried out as previously described^16^. Sprague-Dawley rats were housed under standard conditions at 22 °C with a 12 hour light: 12 hour dark cycle. Female dams were randomly assigned to one of four diets, which they consumed *ad libitum* for 10 days prior to mating and throughout pregnancy and lactation. The diets were either a: control diet (CD; 10% kcal from fat); control diet with CLA (CLA; 10% kcal from fat, 1% total fat as CLA); high fat diet (HF; 45% kcal from fat) and high fat diet with CLA (45% kcal from fat, 1% total fat as CLA) (Research Diets, New Brunswick, USA). The CLA supplement comprised of approximately 50% each of *c*9, *t*11 and *t*10, *c*12 CLA isomers (Stepan Lipid Nutrition, Maywood, USA). At postnatal day 2 (P2), litters were randomly adjusted to 8 pups (4 male, 4 female) to standardise nutrition until weaning. Male and female offspring were weaned onto a standard chow diet (2018 Teklad Global 18% Protein Rodent Diet). At P150, male offspring were fasted overnight, anesthetised with pentobarbitone (intraperitoneal injection; 60 mg/kg) and killed by decapitation. Tissues were dissected, weighed and snap-frozen or fixed in 10% formalin for subsequent analysis. Trunk blood was collected in EDTA vacutainers (Becton Dickinson, Franklin Lakes, USA) and plasma was stored at −20 °C until analysis. Due to the divergent programming effects based on offspring sex, data presented in this manuscript are for adult male offspring (from *n* = 5–6 independent litters).

### Dual-energy X-ray absorptiometry (DXA) scans

DXA scans were performed on adult male offspring at approximately P135. Animals were anesthetised by gaseous isoflurane. Body composition (fat and lean mass) was assessed by DXA with dedicated small animal software (Lunar Prodigy, Madison, USA).

### Oral glucose tolerance tests (OGTTs)

OGTTs were performed on adult male offspring at approximately P142. OGTTs were performed by administering an oral gavage of glucose (2 g/kg body weight) following an overnight fast. Tail blood samples were analysed for glucose concentrations at baseline and 5, 10, 20, 30, 60, 90 and 120 minutes post-gavage by glucose monitor (Optium, Abbott Laboratories, Alameda, CA, USA). Plasma was collected at 0, 20 and 60 minutes post-gavage for insulin analysis.

### Isolation of primary cells from the adipose tissue

Gonadal adipose tissue was finely minced and digested in Krebs ringer bicarbonate buffer with collagenase (2 mg/ml; Gibco by Life Technologies, Auckland, New Zealand). The mixture was incubated in a shaking water bath at 37 °C for 45 minutes, and then filtered. Flow through was centrifuged for 5 minutes at 1700 rpm. The resulting stromal vascular fraction (SVF) pellet was stored in TRI Reagent at −80 °C until subsequent gene expression analysis.

### Plasma analysis

Plasma was analysed for insulin and leptin by commercially available rat-specific ELISA (Crystal Chem, Chicago, USA). The inflammatory cytokines interleukin (IL)-1β, IL-10 and monocyte chemoattractant protein 1 (MCP1) were measured by the Millipore Rat Cytokine Multiplex (Merck Millipore, Darmstadt, Germany). Plasma was also analysed for fasting glucose, free fatty acids, triglycerides, alanine aminotransferase (ALT), aspartate aminotransferase (AST), lipase, how-density lipoprotein cholesterol (LDL), high-density lipoprotein cholesterol (HDL) and total cholesterol by Hitachi 902 autoanalyzer (Hitachi High Technologies Corporation, Tokyo, Japan).

### Gene expression

RNA was isolated from the retroperitoneal adipose tissue, liver and SVF with TRI Reagent following manufacturer’s instructions (Sigma Aldrich, St. Louis, USA). RNA was reversed transcribed using the High-Capacity cDNA Reverse Transcription Kit (Applied Biosystems, Warrington, UK). qPCR was performed with TaqMan Fast Advanced Master Mix and TaqMan Gene Expression Assays (Supplemental Table 1) using either the ABI 7900HT Fast Real-Time PCR System or QuantStudio 6 Flex Real-Time PCR System (Applied Biosystems, Warrington, UK). To control for variability between samples, genes of interest were normalised to at least two reference genes (peptidylprolyl isomerase A (*Ppia*), hypoxanthine phosphoribosyltransferase 1 (*Hprt1*) and/or glyceraldehyde 3-phosphate dehydrogenase (*Gapdh*)). The comparative C_T_ method (2^−ΔΔCT^) was used to analyse data^49^. Details of TaqMan gene expression assays are presented in Supplementary Table 1.

### Protein expression

Retroperitoneal adipose tissue was homogenised in ice cold RIPA buffer (#20-188; Merck Millipore, Darmstadt, Germany) supplemented with protease and phosphatase inhibitor cocktail (Thermo Fisher Scientific, Auckland, New Zealand). Total protein concentration was measured by Pierce BCA protein assay (Thermo Fisher Scientific, Auckland, New Zealand). Samples containing equal amounts of protein were denatured in Laemmli buffer with 100 mM dithiothreitol (Bio-Rad, Auckland, New Zealand) by heating for 5 minutes at 95 °C. Samples were loaded on hand cast 12% gels and separated by sodium dodecyl sulfate polyacrylamide gel electrophoresis (SDS-PAGE). Protein was transferred onto polyvinylidene fluoride (PVDF) membranes using the Trans-Blot Turbo Transfer System (Bio-Rad, Auckland, New Zealand). Membranes were incubated with the following primary antibodies overnight at 4 °C with gentle agitation: PPARγ (1:2000; ab209350, Abcam, Melbourne, Australia), CCAAT-enhancer-binding protein α (C/EBPα, 1:1000; #2295 Cell Signaling Technology, Danvers, USA) and β-actin (1:20000; A2228, Sigma-Aldrich, St. Louis, USA). Proteins were detected with Amersham ECL Select Western Blotting Detection Reagent (GE Healthcare Life Sciences, Auckland, New Zealand) on the ChemiDoc MP System (Bio-Rad, Auckland, New Zealand). Band densitometry analysis was performed with ImageJ 1.48 v software (US National Institutes of Health, Bethesda, USA).

### Histological analysis

Retroperitoneal adipose tissue samples were fixed in 10% neutral buffered formalin (*n* = 10/group) and were paraffin embedded and sectioned (5 μm) with a Leica RM 2135 rotary microtome (Leica Instruments, Nussloch, Germany). Standard hematoxylin and eosin staining was performed and sections were mounted with DPX mountant. Slides were viewed under light microscope and images were captured with NIS Elements-D software (Nikon 800, Tokyo, Japan). Four representative images were taken from each section. Images were blindly analysed in ImageJ to determine adipocyte size.

### Statistical analysis

Statistical analysis was performed using SigmaPlot 12.0 (Systat Software Inc., San Jose, USA). Curves were analysed by repeated measures two-way ANOVA, with maternal diet and time as factors. All other data were analysed by two-way factorial ANOVA, with maternal HF diet and maternal CLA supplementation as factors. *Post-hoc* Holm-Sidak tests were performed where indicated for multiple comparisons testing between groups. Differences between groups were considered significant at *P* < 0.05. All data are presented as means ± SEM.

## Electronic supplementary material

Supplementary information
