# Supplementary material for: Conjugated Linoleic Acid Supplementation Improves Maternal High Fat Diet-Induced Programming of Metabolic Dysfunction in Adult Male Rat Offspring
Source: Sci Rep. 2017 Jul 27;7:6663. doi: 10.1038/s41598-017-07108-9 (PMC5532367; doi:10.1038/s41598-017-07108-9)

Supplemental Data

Conjugated Linoleic Acid Supplementation Improves Maternal High Fat Diet-Induced Programming of Metabolic Dysfunction in Adult Male Rat Offspring

Stephanie A Segovia, Mark H Vickers, Clint Gray, Xiaoyuan D Zhang, Clare M Reynolds

Supplemental Table 1: TaqMan Gene Expression Assays. (Applied Biosystems, Warrington, UK).

Gene Abbreviation	Gene Name	Assay ID
Abca1	ATP binding cassette subfamily A member 1	Rn00710172_m1
Abcg8	ATP binding cassette subfamily G member 8	Rn00590367_m1
Arg1	arginase 1	Rn00691090_m1
Cd11c	integrin subunit alpha X (Itgax)	Rn01511082_m1
Cd36	cluster of differentiation 36 (fatty acid translocase)	Rn02115479_g1
Cd68	cluster of differentiation 68	Rn01495634_g1
Cpt1a	carnitine palmitoyltransferase 1A	Rn00580702_m1
Dgat1	diacylglycerol O-acyltransferase 1	Rn00584870_m1
Dlk1	delta like non-canonical notch ligand 1	Rn00587011_m1
Fasn	fatty acid synthase	Rn01463550_m1
Glut2	glucose transporter type 2	Rn00563565_m1
Il-10	interleukin 10	Rn00563409_m1
Il-1β	interleukin 1β	Rn00580432_m1
Il-6	Interleukin 6	Rn01410330_m1
Ldlr	low-density lipoprotein receptor	Rn00598442_m1
Mcp1	monocyte chemoattractant protein 1	Rn00580555_m1
Mrc1	mannose receptor, C type 1	Rn01487342_m1
Pck1	phosphoenolpyruvate carboxykinase 1	Rn01529014_m1
Pgc1α	PPARγ coactivator 1 alpha	Rn00580241_m1
Pparα	peroxisome proliferator-activated receptor α	Rn00566193_m1
Scd1	stearoyl-CoA desaturase 1	Rn00594894_g1
Srebf1	sterol regulatory element binding transcription factor 1	Rn01495769_m1
Tnfα	tumour necrosis factor α	Rn01525859_g1

Supplemental Figure 1: Hepatic gene expression in adult male offspring. qPCR was performed to determine gene expression of markers related to: **(A)** lipid transport and

metabolism; **(B)** inflammation and **(C)** glucose metabolism. Data analysed by two-way ANOVA (*n*=10/group). Data expressed as means ± SEM.

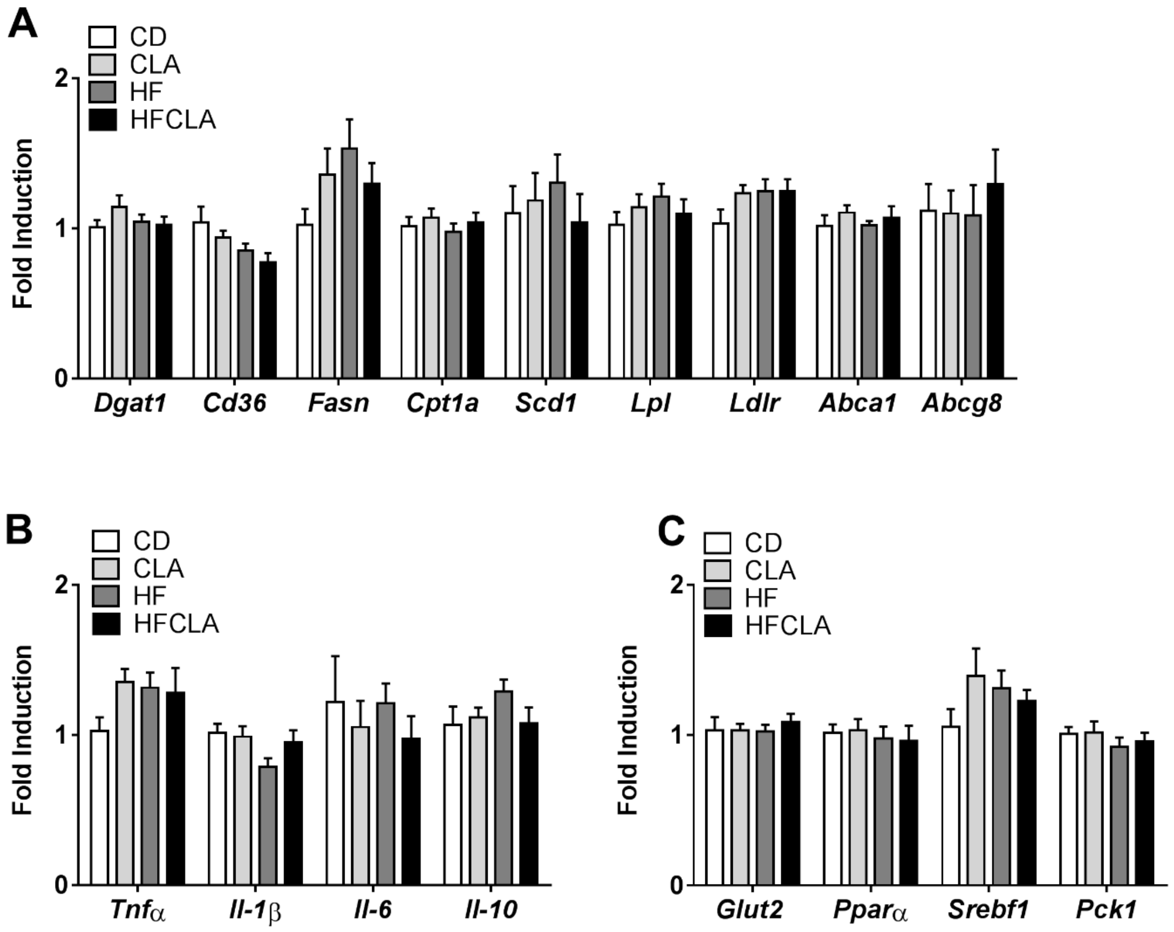

Supplement: Supplementary file 1 — Supplementary information [file 41598_2017_7108_MOESM1_ESM.pdf]
